# Experimental-confirmation and functional-annotation of predicted proteins in the chicken genome

**DOI:** 10.1186/1471-2164-8-425

**Published:** 2007-11-19

**Authors:** Teresia J Buza, Fiona M McCarthy, Shane C Burgess

**Affiliations:** 1 Department of Basic Sciences, College of Veterinary Medicine, Mississippi State University, Mississippi State, MS 39762, USA; 2 Institute for Digital Biology, Mississippi State University, Mississippi State, MS 39762, USA; 3 Life Sciences and Biotechnology Institute, Mississippi State University, Mississippi State, MS 39762, USA; 4 Mississippi Agricultural and Forestry Experiment Station, Mississippi State University, Mississippi State, MS 39762, USA

## Abstract

**Background:**

The chicken genome was sequenced because of its phylogenetic position as a non-mammalian vertebrate, its use as a biomedical model especially to study embryology and development, its role as a source of human disease organisms and its importance as the major source of animal derived food protein. However, genomic sequence data is, in itself, of limited value; generally it is not equivalent to understanding biological function. The benefit of having a genome sequence is that it provides a basis for functional genomics. However, the sequence data currently available is poorly structurally and functionally annotated and many genes do not have standard nomenclature assigned.

**Results:**

We analysed eight chicken tissues and improved the chicken genome structural annotation by providing experimental support for the *in vivo *expression of 7,809 computationally predicted proteins, including 30 chicken proteins that were only electronically predicted or hypothetical translations in human. To improve functional annotation (based on Gene Ontology), we mapped these identified proteins to their human and mouse orthologs and used this orthology to transfer Gene Ontology (GO) functional annotations to the chicken proteins. The 8,213 orthology-based GO annotations that we produced represent an 8% increase in currently available chicken GO annotations. Orthologous chicken products were also assigned standardized nomenclature based on current chicken nomenclature guidelines.

**Conclusion:**

We demonstrate the utility of high-throughput expression proteomics for rapid experimental structural annotation of a newly sequenced eukaryote genome. These experimentally-supported predicted proteins were further annotated by assigning the proteins with standardized nomenclature and functional annotation. This method is widely applicable to a diverse range of species. Moreover, information from one genome can be used to improve the annotation of other genomes and inform gene prediction algorithms.

## Background

After genome sequencing, genome annotation is critical to denote and demarcate the functional elements in the genome (structural annotation) and to link these genomic elements to biological function (functional annotation). Structural annotation of newly sequenced genomes begins during the final stages of genome assembly with electronic prediction of open reading frames (ORFs) [1-3]. Sequencing consortiums typically release these predicted genes and their translated products into public databases, where they account for the majority of data for the newly sequenced species [4,5] and are critical for high-throughput wet lab functional genomics (microarray and proteomics) experiments [4,6]. The NCBI Non-Redundant Protein Database (NRPD) and the UniProt Archive (UniParc) do not directly provide functional annotation for these predicted ORFs. The highly curated UniProt Knowledgebase (UniProtKB) database [7] displays functional annotation from the European Bioinformatics Institute Gene Ontology Annotation (EBI-GOA) Project [8], but does not include predicted gene products until there is experimental evidence for their *in vivo *expression. Thus, despite being critical for functional genomics experiments, most data from a newly sequenced genome does not have even preliminary functional annotation. This problem is exacerbated as other public resources such as Ensembl [9]. Entrez Gene [10] and Affymetrix Netaffx [11] use data from UniProtKB or the EBI-GOA Project as their functional annotation source.

GO has become the de facto standard for functional annotation [12]. Annotations are attributed to sources (e.g. a PubMed ID) and to the type of evidence used to make the association (indicated by evidence codes; Table 1). Many of the evidence codes describe direct species-specific experimental evidence such as "inferred from direct assay" (IDA), "physical interaction" (IPI), "mutant phenotype" (IMP) or "genetic interaction" (IGI). Other evidence codes refer to indirect lines of evidence such as functional motifs and structural or sequence similarity. However, by definition, there can be no direct experimental evidence available for determining the function of predicted gene products. Instead, adding GO annotations based upon indirect evidence such as "inferred from electronic annotation" (IEA) or "inferred from structural/sequence similarity" (ISS) provide the first significant and valuable increases in the breadth of annotations for functional modelling.

**Table 1:** Gene Ontology evidence codes

**Code**	**Description**	**Example**
**Direct experimental evidence codes**		
**IDA**	Inferred from Direct Assay	enzyme assays
		*in vitro *reconstitution
		immunofluorescence
		cell fractionation
		physical interaction/binding assay
**IGI**	Inferred from Genetic Interaction	"traditional" genetic interactions such as suppressors, synthetic lethals, etc.
		functional complementation
		rescue experiments
		inference about one gene drawn from the phenotype of a mutation in a different gene
**IMP**	Inferred from Mutant Phenotype	any gene mutation/knockout
		overexpression/ectopic expression of wild-type or mutant genes
		anti-sense experiments
		RNAi experiments
		specific protein inhibitors
		polymorphism or allelic variation
**IPI**	Inferred from Physical Interaction	2-hybrid interactions
		co-purification
		co-immunoprecipitation
		ion/protein binding experiments
**IEP**	Inferred from Expression Pattern	transcript levels (e.g. Northerns, microarray data)
		protein levels (e.g. Western blots)

**Indirect evidence codes**		

**NAS**	Non-traceable Author Statement	Database entries that don't cite a paper
**TAS**	Traceable Author Statement	original experiments are traceable through that article
**IC**	Inferred by Curator	inferred by a curator from other GO annotations
**IGC**	Inferred from Genomic Context	operon structure
		syntenic regions
		pathway analysis
		genome-scale analysis of processes
**NR**	Not Recorded	used for annotations done before curators began tracking evidence types, not used for new annotations
**ND**	No biological Data available	"unknown" molecular function, biological process, cellular component
**IEA**	Inferred from Electronic Annotation	"hits" in sequence similarity searches, if they have not been reviewed by curators; transferred from database records, if not reviewed by curators
**ISS**	Inferred from Sequence or Structural Similarity	sequence similarity (homologue of/most closely related to)
		recognized domains
		structural similarity
		Southern blotting
		protein features, predicted or observed (e.g. hydrophobicity, sequence composition)
**RCA**	Inferred from Reviewed Computational Analysis	predictions based on large-scale experiments (e.g. genome-wide two-hybrid)
		predictions based on integration of large-scale datasets of several types
		text-based computation (e.g. text mining)

Although most GO annotations for newly sequenced species are the IEA-based annotations provided by the EBI-GOA Project [8], these IEA annotations do not initially include the gene products predicted during sequence assembly. Moreover, while IEA annotations are based on functional motifs and sequences, the most rigorous way of assigning function when there is no direct experimental evidence available, is based on strict orthology. Orthology is one of the central concepts of comparative genome analysis. By definition orthologs are genes or proteins in two or more species that share significant similarity, and are thought to have diverged from a common ancestral gene that existed in their last common ancestor [13-17]. Since orthologous pairs have minimum level of evolutionary separation between them, they are more likely to retain a common function. Determination of orthology relations assists knowledge transfer between species and can be used to improve both structural and functional annotation in organisms that have less annotation.

A number of ortholog prediction methods and search tools are available [9,18-20]. However, the number of proteins from one species that is considered to be part of the same orthologous group varies from one method to another due to different algorithms employed and species included in the methods [14]. For example, Homologene [21] does orthology analyses by comparing protein sequences using the BLASTP tool and then matching the sequences using phylogenetic trees built from sequence similarity and synteny, where possible. Ensembl [9] first uses BLASTP and the Smith-Waterman algorithm to identify putative orthologs by reciprocal BLAST analysis and synteny evidence. Inparanoid [17] is based on pairwise similarity scores and it detects best-best hits between sequences from two different species to form the main orthologous group to which other sequences (in-paralogs) are added only if they are closely related. Treefam (Tree families) [18] uses phylogeny based on Ensembl datasets and clusters genes (and corresponding gene products) from multiple organisms into groups that are all descended from a single ancestor gene. In order to obtain good coverage and reliable predicted orthologs, various methods should be integrated [13].

Comparative genome analysis also requires standardized nomenclature. By identifying orthologs of experimentally supported proteins, standardized nomenclature can be added. Committees for standardized nomenclature exist for human and mouse gene and gene products [22] and chicken researchers have followed suit [23] and will use human nomenclature for orthologous chicken genes.

In this work we analysed nine chicken tissues using a three-stage combined high throughput proteomics and computational biology approach to derive "expressed protein sequence tags" (ePSTs) to improve structural annotation by experimentally supporting the *in vivo *expression of computationally predicted chicken proteins [24]. We then used orthology to add standardized gene nomenclature and GO annotations (by transferring functional annotations based on direct experimental evidence for corresponding human and mouse orthologs).

## Results

### Identification of predicted proteins

**Figure 1:**
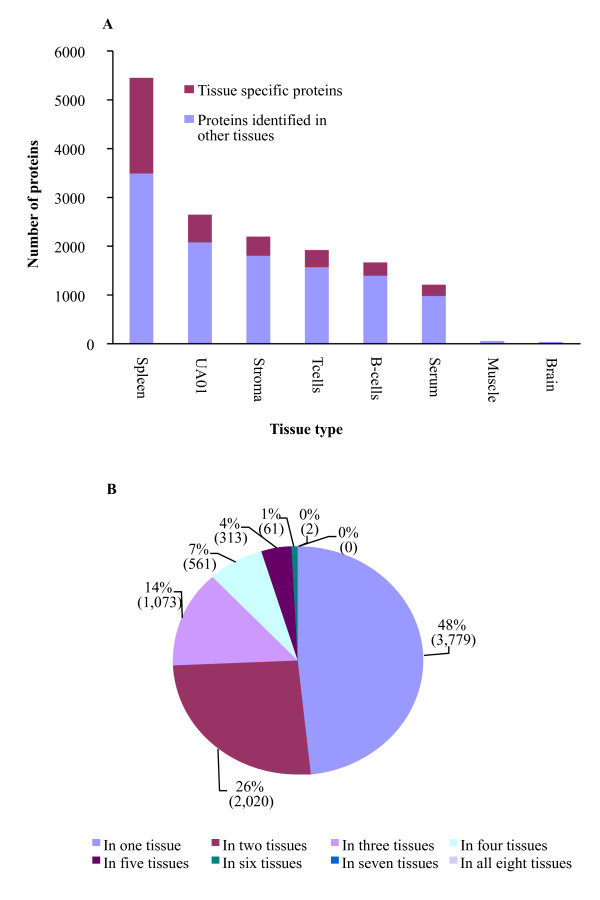
**Chicken *predicted *proteins identified from different tissues**. Proteomic based analysis was used to demonstrate the *in vivo *expression of electronically predicted chicken proteins. (A) The number of predicted chicken proteins identified from each tissue, with the proportion of proteins that were identified in more than one tissue indicated. (B) The majority of proteins were identified in more than one tissue.

In total, we identified 7,809 proteins from the analyzed tissues (see additional file 1), corresponding to 51% of the chicken predicted proteins in NCBI (01/08/2007). In doing so, we also obtained data about the tissue expression patterns of these proteins (Figure 1A). By setting P ≤ 0.05 as a threshold for peptide identification we were able to identify 48,583 peptides that had scores above the threshold in the real database and 438 in the reversed database, giving a peptide false discovery rate (FDR) of 0.9% on the real database. The protein FDR was 1%, equivalent to 78 proteins from this dataset. This FDR is better than recently reported rates [25] and although 4,567 (58%) of the protein identifications in this study were based on single-peptide matches, the low FDR provides a high degree of confidence in these identifications. In other studies, nearly 98% of proteins identified by a single peptide match have been predicted to be correctly identified [26]. Moreover, 44% of the single-peptide matches were identified independently in more than one tissue, providing further evidence for their *in vivo *expression. Interestingly, we identified 30 proteins that were only electronically predicted or hypothetical translations in human.

Not surprisingly, more predicted proteins were identified by mass spectrometry when Differential Detergent Fractionation (DDF) was used as the method for protein isolation, as previously reported [27]. This means that muscle and brain tissues, two tissues which would normally be expected to have the highest number of identified proteins, had the fewest predicted proteins (61 and 36, respectively). We found that 52% of the identified proteins were expressed in more than one tissue (Figure 1B), and their independent identification in multiple tissues lends validity to their *in vivo *expression in chicken. The protein identification and mass spectrometry data has been submitted to the PRoteomic IDEntifications database (PRIDE; [28]), accession numbers 1621–1626, 1654 & 1655.

### ID mapping

One of the most time consuming tasks in high-throughput experiments is navigating among different database identifiers. To assist researchers with their data analysis and facilitate data sharing we mapped all identified proteins to UniParc, IPI (International Protein Index), Entrez Gene and Ensembl identifiers (see additional file 2). Only 80% of the identified proteins were mapped to Ensembl IDs. This may be because Ensembl has a different gene prediction method [9] to that of NCBI and not all of the NCBI predicted proteins are represented in Ensembl.

### Ortholog identification

**Figure 2:**
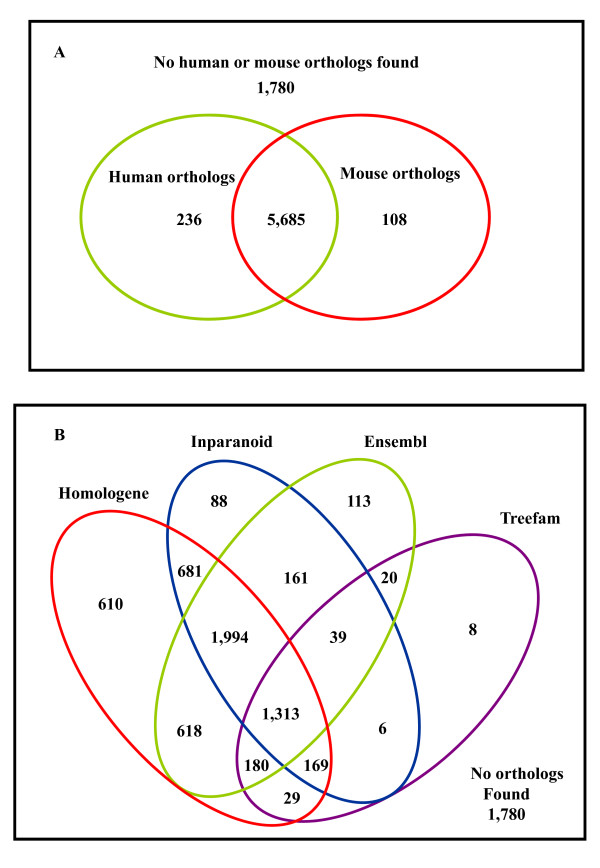
**Chicken – human/mouse orthologs**. **(A) **The number of identified *predicted *proteins that had either human or mouse 1:1 orthologs. **(B) **Distribution of orthologs identified by different orthology prediction methods. The 4 most commonly used ortholog prediction tools are Homologene, Ensembl, InParanoid and Treefam. Human/mouse orthologs were identified for 77% of the identified chicken proteins (see additional file 3).

We identified human or mouse orthologs for 77% (6,008) of the identified chicken predicted proteins (Figure 2A) and 86% of these orthologs are predicted by more than one ortholog prediction method (Figure 2B). Since each of these tools use different methods for ortholog prediction, orthologs predicted by more than one method are more likely to be accurately predicted.

### Standardized nomenclature

The use of standardized nomenclature facilitates comparative biology and aids modelling of functional genomics data. We assigned 5,064 (65%) chicken predicted proteins with HGNC (Human Genome Organization (HUGO) Gene Nomenclature Committee) approved gene symbols and names based on their human or mouse orthologs (see additional file 3). Although it has been agreed to base chicken gene nomenclature on human nomenclature guidelines [23] it is only relatively recently that there has been a concerted effort to provide standardized nomenclature for chicken genes, and the majority of chicken gene products are not named according to standardized nomenclature guidelines. We have assigned standardized nomenclature to chicken genes on a large scale as part of a high-throughput experimental annotation effort.

### Functional Annotation

**Figure 3:**
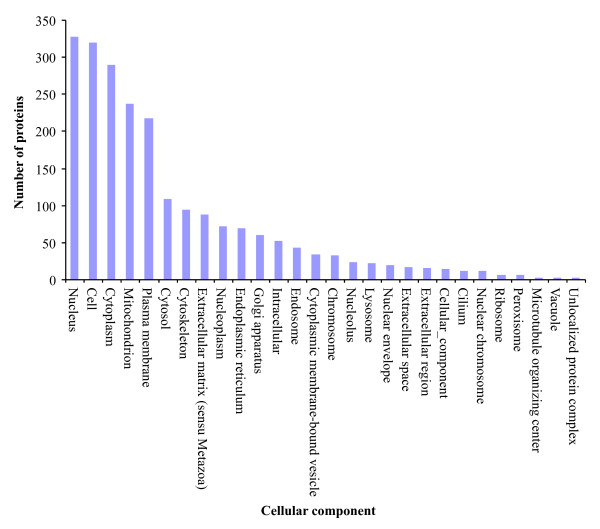
**Overview of cellular component transferred to orthologous chicken predicted proteins**. The GO annotations are summarized to broad terms of cellular component. These GO annotations are publicly available via the AgBase database [4].

**Figure 4:**
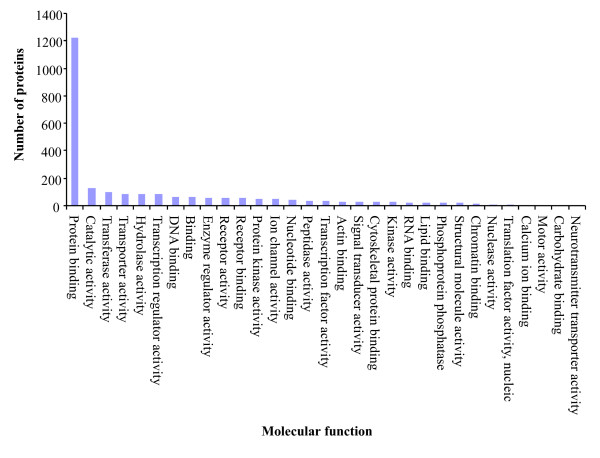
**Overview of molecular function transferred to orthologous chicken predicted proteins**. The GO annotations are summarized to broad terms of molecular function. These GO annotations are publicly available via the AgBase database [4].

**Figure 5:**
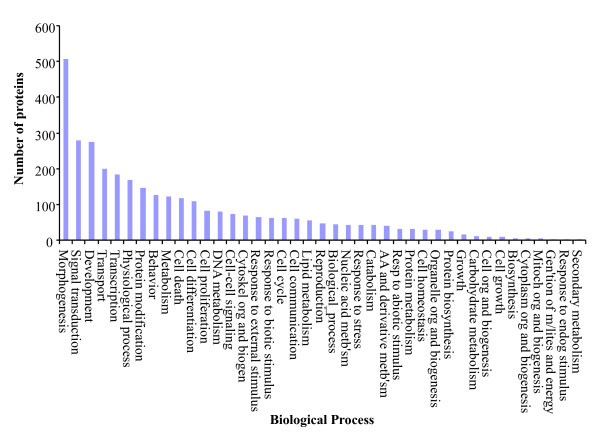
**Overview of biological processes transferred to orthologous chicken predicted proteins**. The GO annotations are summarized to broad terms of biological processes. These GO annotations are publicly available via the AgBase database [4].

To functionally annotate the *predicted *proteins we mapped them to the GO annotations for human and mouse orthologs that are based on direct experimental evidence codes (Table 1). We GO annotated 1,651 (21%) chicken *predicted *proteins with 8,213 associations. These GO annotations are summarized based on cellular component (Figure 3), molecular function (Figure 4) and biological process (Figure 5). These GO annotations represent an increase of 8% over the current chicken GO annotations (EBI-GOA, 04/25/2007) and a doubling of chicken non-IEA annotations. These GO annotations are publicly available via the AgBase database [5] and will enter the pipeline to be submitted to the EBI-GOA Project.

## Discussion

Here we demonstrate a combined approach to provide experimental-based structural annotations and functional annotations based on orthology. The workflow we have developed relies on using proteomics to survey a range of tissues from the species of interest. Newer structural annotation pipelines include the use of ESTs and mRNA in their computational models. We are proposing an analogous method that would include experimental support at the protein level while providing information that can be used to improve structural annotation in the species being studied, provide information to improve annotation in other species and be used to improve open reading frame prediction algorithms. In addition, providing information about tissue specificity and preliminary functional information based on sequence analysis will facilitate analysis of future functional genomics studies.

The chicken genome was sequenced because of its importance as a non-mammalian vertebrate model, its use as a biomedical model to study embryology and [29,30] development and its agricultural importance. A major step that follows after genome sequencing is structural and functional annotation (denoting and demarcating the functional elements in the genome and link these genomic elements to biological function, respectively). When we began the work described in this manuscript only 53% of chicken proteins were known to be expressed *in vivo*, with the remainder being electronically predicted using in silico methods. Moreover, only 52% of chicken gene products had any GO annotations and, although genes predicted during genome assembly may be the bulk of the data for a newly sequenced species, these predicted gene products are not automatically assigned any GO annotation.

The parameters we have used in this study provide strong support for protein expression *in vivo*. In particular, the parameter DeltaCn is a measure of specificity of the match within the database used and a DeltaCn value 0.1 ensures that a peptide is distinctly different from other peptides within the same database. However, a single peptide match to a predicted protein does not necessarily provide evidence that the annotation for the entire open reading frame is accurate; this can only be confirmed by accumulating more mass spectra data and accounting for the detectable peptides within the genome [31]. While some of the predicted proteins we identified were identified on the basis of a single peptide, 44% of these proteins were expressed in more than one tissue, providing additional evidence for their *in vivo *expression. In a typical proteomics experiment 20–67%-of proteins are identified by a single peptide match [26,32,33]. Calculation of false discovery rate has been used to validate peptide or proteins identifications [32,34-37], including proteins identified by a single peptide match. In one study, 90% of the proteins identified by a single peptide were validated by immunoassay detection [33].

By analysis of multiple tissues we maximize the number of predicted proteins identified and provide tissue expression data for these identified proteins. Also, identifying predicted proteins in more than one experiment (52% of the chicken proteins identified were detected in more than one tissue) provides additional confidence that the predicted protein is expressed *in vivo*. In addition, 30 proteins were only electronically predicted or hypothetical translations in human. Identifying these proteins in chicken is additional information to support, not only the expression of these proteins in chicken but also in human based on orthology.

The least number of proteins were identified from the muscle and brain tissues. However, this does not necessarily reflect the biological complexity of these tissues but is more likely a reflection of the different protein extraction method used for these two tissues and amount of sample analyzed.

In addition to providing experimental support for the *in vivo *expression of chicken predicted proteins, we used strict 1:1 orthology with human and mouse genes to provide the identified proteins with standardized gene nomenclature based on established nomenclature guidelines and functional annotations based on the best available data. Since by definition predicted proteins have no direct experimental evidence, assignation of GO annotation for these proteins can be done using either IEA or ISS. While IEA is provided for a large range of organisms by the EBI-GOA Project, this annotation effort does not include predicted proteins and IEA annotations tend to be broad descriptions of function (e.g. "protein binding"). The most rigorous way to assign function in the absence of direct experimental evidence is by strict orthology.

Orthologs are genes in different species that evolved from a common ancestral gene by speciation. Orthologs are, by definition, more likely to share functional similarity [38] and orthology can be used to reliably infer function to their co-orthologs. We determined chicken orthologous genes that pair with human and mouse genes. Since there is no a 'gold standard' method for orthologs identification [14], we integrated different published orthology identification methods that could possibly increase the breadth of orthologs identified. We were able to identify human or mouse orthologs for 77% of the identified chicken proteins. This figure, however, is better than the number that could have been obtained when using only one method (see additional file 3). For example from the total number of identified chicken predicted proteins (7,809), only 71%, 57%, 57% and 23% could have been identified by Homologene, Inparanoid, Ensembl and Treefam, respectively. Each of these methods use different procedures and orthologs identified by more than one method have been reported to be more consistent and reliable [14].

In addition to the experimentally supported predicted proteins that have human or mouse orthologs, there are a further 1,780 predicted proteins that we identified in this study. We are in the process of providing GO functional annotation for these proteins based on sequence similarity to other GO annotated gene products and functional motifs and domains and this information will be also be made publicly available.

Standardized nomenclature is becoming increasingly important with the large amounts of data released by sequencing projects, gene expression microarrays and proteomics. This information will facilitate comparative and functional genomics studies in both avians and mammals. Moreover, assigning functional annotation based on orthology is more robust than using sequence similarity alone [14]. This is because the higher level of functional conservation between orthologous proteins makes orthology highly relevant for protein function prediction. Thus our 8% increase in chicken GO annotated proteins is a significant improvement.

## Conclusion

We demonstrate the value of proteomics to experimentally support the in-vivo expression of electronically predicted proteins of a newly sequenced genome. We assigned standardized nomenclature and GO functional annotations for these newly confirmed proteins. The approach we have developed facilitates comparative and functional genomics studies and may be applied to improve the annotations of a diverse range of newly sequenced genomes.

## Methods

### Tissues and protein extraction

Proteins were isolated from several different tissues in a series of experiments. Bursal B cells and stromal cells were isolated from bursas collected from five 21-day-old Ross 508 mixed sex chickens, muscle from the Pectoralis Major muscle of six 42 day old female chickens, brain from six 42 day old female chickens, spleen from eighteen 7- and 8-day-old advanced intercross Fayoumi and Leghorn mixed sex chickens, T cells from peripheral blood mononuclear cells (PBMC) obtained from adult Ross 508 mixed sex chickens, serum from 20-day-old Ross 508 male chickens. The disease virus-transformed cell line, MDCC-UA01 (obtained from Dr M. Parcells, University of Delaware) was grown as described [39]. Proteins were isolated using Differential Detergent Fractionation (DDF) [27] for each of the tissues except muscle and brain. For the muscle and brain samples, the samples were immediately frozen at -80°C. The samples were then allowed to warm to -21°C and solubilized in lysis buffer (7 M urea, 2 M thiourea, 4% CHAPSO, 8 mM PMSF) with repetitive pulsed sonication on ice. Note that the DDF method has been shown to yield more proteins than a single step lysis of tissues (as used for muscle and brain) [27].

### Proteomics

All solubilized proteins were identified by 2-dimensional liquid chromatography tandem mass spectrometry (2-DLCMS/MS) exactly as previously described [24,27]. Briefly, protein mixtures are trypsin digested and the peptides desalted prior to strong cation exchange followed by reverse phase liquid chromatography coupled directly in line with ESI ion trap MS. A flow rate of 3 μL/min was used for both SCX and RP columns. A salt gradient was applied in steps of 0, 5, 10, 15, 20, 25, 30, 35, 40, 45, 50, 57, 64, 71, 79, 90, 110, 300, and 700 mM ammonium acetate in 5% ACN, 0.1% formic acid and the resultant peptides loaded directly into the sample loop of a 0.18 × 100 mm BioBasic C18 reverse phase liquid chromatography column of a Proteome X workstation (ThermoElectron). The reverse phase gradient used 0.1% formic acid in ACN and increased the ACN concentration in a linear gradient from 5% to 30% in 30 min and then 30% to 65% in 9 min followed by 95% for 5 min and 5% for 15 min.

A database containing only chicken proteins that have been electronically predicted was prepared by parsing the chicken RefSeq entries (chicken gene build 2.1, 01/08/2007) for records with an XP prefix (14,676 proteins). The XP prefix is used to indicate proteins that have been predicted using the GNOMON pipeline. Redundancies were minimized by using the RefSeq dataset rather than the dataset from the Non-redundant Protein Database. The RefSeq database contained 19,500 chicken proteins but only the 14,676 GNOMON predicted proteins were used in this study. Trypsin digestion was applied in silico to the predicted protein database including mass changes due to cysteine-carboxyamidomethylation and methionine oxidation.

The MS2 spectra were then used to search the non-redundant predicted protein database using Cluster 3.2 (Bioworks Browser 3.2, Thermo Electron, San Jose, CA). The peptide (MS precursor ion) mass tolerance was set to 1.4 and the groups scan to 1.0. Peptide molecular range was set to 600–3500. Only peptides ≥ 6 amino acids in length that had cross correlation (Xcorr) scores of 1.5, 2.0 and 2.5 (for +1, +2, and +3 charge state, respectively) and DeltaCn of > 0.1 [25,40,41] were considered matches. To quantify the peptide false discovery rate (FDR), we used the reverse database function in Bioworks 3.2 to search all MS2 spectra against a reversed version of our predicted proteins database using the same search criteria described above. Prior to calculating the FDR, we calculated the probability of each peptide match from both real and reversed database based on the product of XCorr and DeltaCn and set a cut-off of P ≤ 0.05 for individual peptide identifications. With this probability as the cut-off, we calculated the FDR using the expected proportion E(V) of incorrect identifications from correct identifications (R) [36]: FDR = E(V)/R. Proteins were identified based on the peptides that pass the above criteria.

### ID Mapping

Proteins identified by SEQUEST search algorithm have a Genbank identifier (gi) and RefSeq identifiers. In order to facilitate data sharing with public databases and ortholog determination we mapped the identified proteins to corresponding identifiers from UniProt Archive (UniParc), the International Protein Index (IPI), Entrez Gene and Ensembl protein identifiers using either different online tools for ID mapping [42-45] or an in-house Perl script (MapProtID.pl) to match different ID datasets. In cases where the program could not find an identifier, we used gi or RefSeq numbers to manually search co-identifiers in the UniParc [46], IPI [47], Entrez [48] or Ensembl [49] databases.

### Ortholog Prediction

Chicken-human orthologs were downloaded from the HGNC (Human Genome Organization (HUGO) Gene Nomenclature Committee) Comparison of Orthology Predictions (HCOP) site [50] using the HCOP search tool [20,51]. HCOP integrates and displays the orthology assertions made by different ortholog prediction methods such as Ensembl [9], Homologene [21,52], Inparanoid [17], MGI (Mouse Genome Informatics) [53] and Treefam [18]. In cases where we could not identify chicken-human orthologs we manually checked Homologene [52], Inparanoid [54] or Ensembl [49,55] in order to obtain the most recent data. Chicken-mouse orthologs were downloaded only from Homologene, Inparanoid and Ensembl because HCOP does not predict chicken-mouse orthologs

### Standardized Nomenclature

Standardized gene nomenclature is vital for effective scientific communication [22] and chicken researchers have agreed to use human nomenclature for orthologous chicken genes [23]. In this study we assigned chicken standardized nomenclature based on HGNC approved gene symbols and names that were associated with the human or mouse orthologs. We manually check the existence of each symbol and name in the HGNC nomenclature database before transferring it to chicken. In cases where the human or mouse gene symbol or name was not found or withdrawn from HGNC, no symbol or name was assigned to the chicken co-ortholog. To distinguish chicken from human genes the symbol assigned to chicken gene products are all in lowercases except for the first letter, as is the convention for mouse.

### Functional Annotation

Since orthologs are presumed to have the same function, useful functional information can be extracted from other species when annotating orthologous gene products with unknown functions. To provide GO annotation for the identified chicken predicted proteins, we downloaded the human and mouse GO annotations from either the European Bioinformatics Institute GO annotation project (EBI-GOA: 03/12/2007) or searched Ensembl [49] using Biomart [43,55]. We assigned the chicken predicted proteins the GO annotations of human and mouse orthologs that are only based on direct experimental evidence codes (Table 1) and each chicken GO annotation was assigned an ISS GO evidence code, as per usual GO annotation procedure.

### Public Availability of Data

Experimentally supported predicted proteins will be shared with the NCBI database, standardized nomenclature made available to both the NCBI and UniProt databases and GO annotations made available publicly via AgBase, the EBI-GOA Project and the GO Consortium. Assigned GO annotations are publicly available via the AgBase database [5] and will be submitted to the EBI-GOA Project. A summary of these GO annotations was obtained by mapping the associated GO terms to the Generic GOSlim Sets [56] using GOSlimViewer [4,5].

## Authors' contributions

TJB contributed in the data generation, analysis of results and writing the draft of the manuscript. Both FMM and SCB contributed in the formulation, design of the study and manuscript preparation. All authors read and approved the final manuscript.

## Supplementary Material

Additional file 1 **Proteins identified by DDF-MudPIT and their distribution by tissue type**. Column 1 shows the RefSeq numbers of the identified chicken predicted proteins, column 2 indicates the corresponding predicted protein names (assigned by NCBI). Columns 3–8 shows the different types of tissue/cells used in this study and + and - indicate the presence or absence of the proteins in the specified tissue/cell, respectively. Click here for file

Additional file 2 **Database identifiers for the predicted proteins**. RefSeq and gi identifiers (columns 1 & 2) are cross-referenced with other database identifiers for each of the identified chicken proteins. Click here for file

Additional file 3 **Chicken-human/mouse orthologs predicted by different tools**. Using either the human or mouse orthologs shown in column 3, a standardized gene symbol and name (column 4 & 5) was assigned to 5,064 (65%) of the predicted proteins identified in this study. Columns 6–10 list the orthology prediction tools that were used to predict the human or mouse orthologs. Click here for file
